# The Role of Nutraceuticals in Osteoarthritis Prevention and Treatment: Focus on n-3 PUFAs

**DOI:** 10.1155/2021/4878562

**Published:** 2021-12-10

**Authors:** Francesca Oppedisano, Rosa Maria Bulotta, Jessica Maiuolo, Micaela Gliozzi, Vincenzo Musolino, Cristina Carresi, Sara Ilari, Maria Serra, Carolina Muscoli, Santo Gratteri, Ernesto Palma, Vincenzo Mollace

**Affiliations:** ^1^Institute of Research for Food Safety & Health IRC-FSH, University Magna Graecia, 88100 Catanzaro, Italy; ^2^Nutramed S.c.a.r.l., Complesso Ninì Barbieri, Roccelletta di Borgia, 88021 Catanzaro, Italy; ^3^IRCCS San Raffaele Pisana, Via di Valcannuta, Rome, Italy

## Abstract

Osteoarthritis (OA) is a disease caused by joint degeneration with massive cartilage loss, and obesity is among the risk factors for its onset, though the pathophysiological mechanisms underlying the disease and better therapeutic approach still remain to be assessed. In recent years, several nutraceutical interventions have been investigated in order to define better solutions for preventing and treating OA. Among them, polyunsaturated fatty acids (n-3 PUFAs) appear to represent potential candidates in counteracting OA and its consequences, due to their anti-inflammatory, antioxidant, and chondroinductive effects. PUFAs have been found to counteract the onset and progression of OA by reducing bone and cartilage destruction, inhibiting proinflammatory cytokine release, reactive oxygen species (ROS) generation, and the NF-*κ*B pathway's activation. Moreover, a diet rich in n-3 PUFAs and their derivatives (maresins and resolvins) demonstrates beneficial effects on associated pain reduction. Finally, it has been shown that together with the anti-inflammatory and antioxidant properties of eicosapentaenoic (EPA) and docosahexaenoic (DHA) acids, their antiapoptotic and antiangiogenic effects contribute in reducing OA development. The present review is aimed at assessing evidence suggesting the potential benefit of nutraceutical supplementation with PUFAs in OA management according to their efficacy in targeting relevant pathophysiological mechanisms responsible for inflammation and joint destruction processes, and this may represent a novel and potentially useful approach in OA prevention and treatment. For that purpose, a PubMed literature survey was conducted with a focus on some in vitro and in vivo studies and clinical trials from 2015 to 2020.

## 1. Introduction

Osteoarthritis (OA) is a degenerative joint disease associated with massive cartilage loss [1–3], affecting approximately 15% of the total population, and 60% of the elderly population [4]. Normal articular cartilage is made up of connective tissue and covers the load-bearing surfaces at the ends of long bones [5]. The only cells in cartilage are chondrocytes, which constitute the cartilage extracellular matrix (ECM) through a balance between synthesis and degradation. The ECM is basically made up of collagen type II (COL2A1) and proteoglycans, such as aggrecan. This composition guarantees structural integrity and the absence of friction during joint movement [5, 6]. Below the cartilage in the joint is a dense formation: the subchondral bone plate and trabecular bone in the epiphysis, the function of which is to support the loads applied to the joint. There are numerous vessels in the trabecular portion that provide nourishment to the cartilage. Bone formation is attributable to osteoblasts, while osteoclasts' function is bone resorption. In order to perform their functions, these cells need a continuous supply of adenosine triphosphate (ATP); therefore, they are metabolically very active. Articular joints also contain synovial tissue, which is subdivided into intima (the inner layer) and subintima. The synovial intimal cells are fibroblast-like synoviocytes (FLS) and macrophage-like synoviocytes (MLS); the former is responsible for synovial fluid viscosity, while the latter is tissue-resident macrophages. Moreover, menisci, ligaments, tendons, and adipose depots are all responsible for biomechanical stability and joint function [5]. Hands, knees, and hips are particularly affected by OA, and more specifically, the articular bodies, capsules, bursae, cartilage menisci, ligaments, and muscles [1, 4, 7]. The osteochondral unit consists of cartilage, subchondral bone, and calcified cartilage. This unit, essential for load distribution and joint movement, is modified as OA progresses [1, 2, 4]. In the early stages of disease onset, the cortical plate and subchondral bone undergo rapid bone remodeling, with concomitant bone loss and increased porosity. Changes in calcified cartilage and tidemark destruction are related to the improper passage of substances and vessel generation. Modification of the osteochondral unit leads to unbalanced load distribution with consequent cartilage destruction, which, over time, determines OA onset and progression [1, 2]. Furthermore, alterations in subchondral bone are responsible for the different crosstalks between chondrocytes and bone cells, which contribute to cartilage destruction [1]. Chondrocytes, responsible for bone formation, are damaged by proinflammatory action of interleukins and metalloproteinases [1, 7]. Furthermore, chondrocytes express molecules such as VEGF (vascular endothelial growth factor), MMP-13 (matrix metallopeptidase 13), and RUNX2 (runt-related transcription factor 2), implicated in hypertrophy and differentiation [1, 2, 7]. Hypertrophy and the surrounding calcified extracellular matrix alter the tidemark on the osteochondral interface, causing microcracks and thinning of the cartilage [1, 2]. OA onset is characterized by greater bone remodeling, with simultaneous bone reduction under articular cartilage. Decreased remodeling and subchondral densification occur during disease progression, together with synovial inflammation and increased inflammatory and catabolic responses [1, 2, 8, 9]. In light of this, intervening on subchondral bone remodeling and maintaining osteochondral unit structural integrity could represent a therapeutic strategy in preventing the onset and progression of OA [1].

## 2. Role of Inflammation and Oxidative Stress in OA

The structural changes characterizing OA are determined by a series of factors, the most important of which is inflammation [10]. From a clinical point of view, joint inflammation in OA is characterized by joint swelling, warmth, and pain [2, 10]. In particular, inflammation of the synovial membrane, known as synovitis, occurs as a result of interaction between degraded cartilage fragments and the immune system, which generates a protective inflammatory response via synoviocytes [10]. The cartilage fragments are identified as foreign bodies, and therefore, trigger a response from both the innate and adaptive immune systems. Consequently, the inflammatory response is generated through the activation of inflammatory signaling pathways, such as the NF-*κ*B (nuclear factor-*κ*B) pathway [7, 10]. This change is closely related to aging and also occurs in the absence of other conditions, such as obesity and metabolic syndrome [7]. The most studied inflammatory mediators of OA are cytokines that amplify low-grade inflammation, further compromising cartilage [10, 11]. However, obesity is one of the most important risk factors in the onset of OA [5]. It has been shown that the onset of posttraumatic OA is more linked to biomechanical factors, while metabolic OA arises following chronic inflammation and an unbalanced diet; conditions characteristic of obesity [5, 12]. In fact, chronic inflammation, characterized by the increased synthesis of proinflammatory cytokines, such as IL- (interleukin-) 1*β* and TNF- (tumor necrosis factor-) *α*, determines greater osteoclast activation, which is responsible for bone resorption [5]. Furthermore, hormones such as leptin and visfatin, which are associated with obesity, are also involved in OA onset and progression. It has been shown that knee osteoarthritis is the most common form among obese people and, in particular, among aging adults [12]. Therefore, damage to joints is not only determined by increased body weight but is also mainly due to a greater synthesis of matrix metalloproteinases (MMPs), such as MMP-1, −3, −9, and −13, disintegrin, and metalloproteinase with thrombospondin motifs (ADAMTS), such as ADAMTS-4 and −5, the presence of which is related to ECM degradation, synovitis, and cartilage and bone injuries [3, 5–7, 13]. IL-1*β* induces the upregulation of these enzymes in addition to other catabolic factors, including inflammatory mediators, nitric oxide (NO), prostaglandin E2 (PGE2), cyclooxygenase-2 (COX-2), and reactive oxygen species (ROS) [6, 14–16]. Additionally, oxidative stress can trigger joint inflammation and pain in response to cellular senescence or obesity-related systemic inflammation. In fact, ROS production is deeply involved in OA triggering the inflammation cycles and catabolism, leading to a reduction of glycosaminoglycans and collagen modifications, causing chondrocyte homeostasis alteration and irreversible cartilage matrix degradation with OA development [5–8]. Joint aging and dysfunction are also related to impaired autophagy, which results in chondrocytes losing the ability to maintain homeostasis and survive in pathological conditions. Indeed, in aged cartilage and in mouse joints with surgically induced OA, autophagic protein expression is downregulated [7, 17]. In OA inflammation, NO plays an important pathological role. NO is synthesized in chondrocytes by inducible NO synthase (iNOS), and its high production rate generates an inflammatory state that contributes to cartilage destruction and cell damage [7, 10]. Studies conducted in OA patients have shown greater NO concentration and iNOS expression in chondrocytes compared to the (still high) levels present at the synovial level. Therefore, NO produced at the cartilage level contributes to OA pathogenesis. In particular, a higher iNOS expression has been demonstrated in the superficial area of OA cartilage, which thus pinpoints the commencement of OA damage, i.e., the damaged cartilage is responsible for the greater NO production. The latter causes cartilage destruction, as it increases chondrocyte-mediated matrix degradation, increases MMPs activity and, at the same time, inhibits the synthesis of matrix components, such as COL2A1 and aggrecan. NO contributes to OA pathogenesis by triggering the inflammatory response, along with increased synthesis of PGE2 and inflammatory cytokines [16]. Furthermore, NO can also be involved in mechanisms related to oxidative damage and chondrocyte death by apoptosis. Therefore, iNOS modulation represents a possible target for OA therapy; in fact, the chondroprotective effects of many molecules of plant origin, such as pomegranate extract, have been demonstrated [10].

## 3. Therapies in OA

OA is a disabling joint disease with a multifactorial mechanism, causing major impairment to quality of life, as well as pain, limitation of movement, and disability [2, 4, 10, 17]. To date, there are no effective therapies for OA; just therapies that confer symptomatic relief or a definitive treatment, such as joint arthroplasty [2, 10, 17]. Treatment such as joint arthroplasty still has a high postsurgical chronic pain incidence that ranges between 20% and 40% [18]. Osteoarthritis pain after prosthesis implantation is one of the most severe secondary syndromes, depending not only on surgery but also on organic changes before and after joints replacement [2, 10, 17]. Opioid employment could influence postsurgical pain and lead to tolerance or addiction. It is well known that the involvement in hypersensibility of the immune system, the nervous system, and the peptidergic ones is connected due to the opioid receptors on immune cells surface. Recently, it has been shown that the percentage of Mu-positive B cells is statistically lower in OA patients, and this data could be used as a biological marker for an objective diagnosis of chronic pain [19]. Therefore, in order to be effective, OA therapies must be applied either in a preventive manner, or in the initial stages of disease onset. Chronic joint pain is the main symptom of OA, and strategies to relieve it are necessary to improving the quality of life in patients with OA [14, 20]. In fact, the drugs used in OA patients are analgesic and/or nonsteroidal anti-inflammatories (NSAIDs), which only counteract symptoms without acting on OA progression and pathophysiology [7, 9, 21]. Additionally, in long-term therapy, NSAIDs have side effects at the gastrointestinal, renal, and cardiovascular levels; they also manifest liver toxicity, hemorrhaging, and negative effects on chondrocytes and cartilage matrix formation [7, 9, 22]. Consequently, in recent years, alternative solutions with fewer side effects have been sought by the scientific community, such as treatment with natural compounds. In fact, nutritional treatments for the prophylaxis and therapy of other diseases, including heart disease, hepatic steatosis, and metabolic syndrome, are all considered viable therapeutic alternatives [23–32]. Therefore, such compounds are believed to be important in the prevention and management of articular cartilage structural damage in OA [2, 3, 33]. The data obtained from *in vitro* and *in vivo* preclinical studies confirm the anti-inflammatory and antioxidant effects of the natural compounds used in counteracting both the progression and symptoms in OA [7]. Additionally, human clinical trials have demonstrated the effectiveness of natural compounds for the management and relief of OA pain; this is likely attributable to their anti-inflammatory and antioxidant properties [20]. Thus, although nutraceutical supplementation with natural compounds has been widely used in the past decades to find better solutions to counteract OA development, the benefit of such an approach is not well defined and further studies are required to understand the potential for nutraceutical supplementation in OA treatment. Recently, evidence has been collected showing that polyunsaturated fatty acids (PUFAs) may represent right candidates for nutraceutical supplementation in treating OA, alongside with traditional pharmacological approach. In particular, it has been suggested that their selective anti-inflammatory and antioxidant properties may significantly produce chondroprotective thereby attenuating cartilage loss. In this review, we aim to summarize scientific data demonstrating the effectiveness of PUFAs in OA management and their potential role in nutraceutical supplementation in OA-related pathophysiological mechanisms. The interest in these nutraceuticals is linked to the fact that they represent important components of the Mediterranean diet.

## 4. Polyunsaturated Fatty Acids (PUFAs)

Polyunsaturated fatty acids (PUFAs), and all unsaturated fatty acids (FAs) in general, are lipids consisting of a long hydrocarbon chain with a carboxyl group (-COOH) at the polar hydrophilic end and a nonpolar hydrophobic methyl group (-CH3) at the opposite end [34]. Two classes of PUFAs, n-3 and n-6, are defined as “essential,” as they must be taken in via the diet because humans do not have the *Δ*12- and *Δ*15-desaturases that catalyze double bond formation in positions n-3 and n-6 of the FA carbon chain. In particular, the n-3 PUFAs have their first double bond between the third and fourth carbon atoms, while n-6 PUFAs have it between the sixth and seventh carbon atoms, counting from the methyl end of the FAs [35]. Linoleic acid (LA, 18 : 2, omega-6) and *α*-linolenic acid (ALA, 18 : 3, omega-3) are essential PUFAs as they must be taken in via the diet [36]. The main products of LA metabolism are n-6 PUFA, *γ*-linolenic, and arachidonic acid, while n-3 PUFAs, such as eicosapentaenoic acid (EPA, 20 : 5 n-3) and docosahexaenoic acid (DHA, 22 : 6 n-3), derive from ALA [36, 37]. The vegetable dietary sources of LA are safflower, soy, and corn oils. ALA, on the other hand, is present in flax seeds, beans, nuts, and the leaves of some green plants. The levels of EPA and DHA obtained by the liver from *α*-linolenic acid are minimal, and most of their content in the body derives from the diet. In particular, EPA and DHA are abundant in the flesh of both lean and fatty marine fish, as well as in fish oil and algal-derived supplements, although they can also be found in lower quantities in many other foods of animal origin. Therefore, including them in our daily diet in the correct proportions is highly recommended [34–37].

### 4.1. Role of n-3 and n-6 PUFAs

Western diets (WD) usually have a higher content of n-6 PUFAs than n-3 PUFAs; the n-6/n-3 ratio is usually higher than 15 : 1. The recommended daily intake is around 4 : 15. This unbalanced ratio, in favor of n-6 PUFAs, favors proinflammatory eicosanoid synthesis, causing the onset of inflammatory and autoimmune diseases [38]. EPA and DHA are incorporated into cellular phospholipid membranes, but are also precursors of immune-inflammatory signaling modulators. In particular, EPA and DHA generate prostaglandins, leukotrienes, and D- and E-series resolvins, which have potent anti-inflammatory effects through COX and lipoxygenase (LOX) activity [39, 40]. Conversely, n-6 PUFAs perform proinflammatory roles, regulating various inflammatory processes and genes. Furthermore, arachidonic acid is a precursor to proinflammatory mediating prostaglandins, thromboxanes, and leukotrienes, while linoleic acid causes more severe inflammatory responses [37]. Given the opposite effects of n-3 and n-6 PUFAs on the inflammatory response, their ratio is fundamental to the regulation of inflammatory signaling homeostasis [40]. Numerous epidemiological studies correlate n-3 PUFAs intake with long-term beneficial effects on human health. In fact, the efficacy of n-3 PUFAs in the prevention and treatment of various pathologies, such as cerebral ischemia, non-alcoholic fatty liver disease (NAFLD), cardiovascular diseases, and neurodegenerative diseases, is well known [26, 27, 41–44]. Importantly, several evidences have shown that PUFAs play an important role in OA.

### 4.2. PUFAs and OA

#### 4.2.1. DHA, Bone Remodeling, and Angiogenesis

To better investigate the role of the osteochondral unit in OA onset and progression, an *in vivo* study was conducted in an ACLT-induced rat model, and an *in vitro* one in RAW264.7 (mouse mononuclear macrophage leukemia cells) and HUVECs (human umbilical vein endothelial cells) [1]. In the *in vivo* model, DHA's effect on bone remodeling and vessel formation in the osteochondral unit was investigated. Microcomputed tomography (micro-CT) images of subchondral bone revealed DHA's positive effect on the bone surface to bone volume (BS/BV) and bone volume to tissue volume (BV/TV) ratios, indicating that DHA protects the microstructure of subchondral bone in the initial phase of OA. The reported study showed that DHA inhibits osteoclast differentiation, reducing areas of bone resorption and confirming osteoclasts' role in OA. DHA has been shown to inhibit mRNA and the protein expression of osteoclast markers TRAP (tartrate-resistant acid phosphatase) and CTSK (Cathepsin K), which are involved in bone turnover. Therefore, by inhibiting these protein expressions, DHA inhibits osteoclast differentiation and bone remodeling, along with bone mass preservation. RANKL (receptor activator of nuclear factor kappa-*Β* ligand) is also involved in this process, as its expression is downregulated by the presence of DHA, both in ACLT rats and in cells, thus, preventing cartilage degradation. Furthermore, NFATc1 (nuclear factor of activated T-cells, cytoplasmic 1) is also involved in the terminal osteoclast differentiation process. It is known that NFATc1 and RANKL signaling pathways can perform their functions either in synergy or independently. The study's results for RAW264.7 cells treated with RANKL demonstrate that DHA presence reduces the levels of NFATc1 expression, influencing osteoclast differentiation. Similar results were recorded for the DHA regulation of MITF (microphthalmia-associated transcription factor) expression, a member of the MIT family that is involved in the differentiation process that regulates TRAP mRNA expression levels. In this way, DHA suppresses osteoclast differentiation. In addition, an increase in angiogenesis was found in OA development. It was also shown that DHA slows OA progression by reducing angiogenesis at the interface between subchondral bone and calcified cartilage, inhibiting the proliferation and migration of HUVECs. DHA blocks the VEGF–VEGFR2 signaling pathway, responsible for blood vessel formation, which is particularly active in OA models. Therefore, DHA slows down cartilage degeneration.

#### 4.2.2. The n-6/n-3 PUFA Ratio and MMP-13 Expression

Since MMP-13 expression increases in OA chondrocytes, the effects of different n-6/n-3 PUFAs ratios were studied both *in vitro* (in inflammatory human chondrocytes) and in Sprague-Dawley rats with arthritis induced by Freund's complete adjuvant, treated for six weeks [45]. In particular, the effects of LA/ALA ratios ranging from 1 : 1 to 10 : 1 were tested. The results obtained *in vitro* and *in vivo* studies showed no variation in cell proliferation, but an LA/ALA ratio of 1 : 1 is more effective in reducing mRNA and MMP-13 protein levels. In particular, the serum MMP-13 and IL-1 levels are lower when treated with LA/ALA ratios of 1 : 1 and 2 : 1. The same LA/ALA ratios reduced the paw swelling rate, and the histological analysis reported less cartilage damage. Therefore, this study demonstrates that an equal ratio of n-6 and n-3 PUFAs is the most effective in inhibiting MMP-13 expression and induced arthritis in rats, thus, representing a good approach for the treatment of OA symptoms. It is also known that MMP-13 is responsible for COL2A1 degradation and destruction, a condition that leads to OA onset and progression. COL2A1 synthesis is also inhibited by IL-1*β*, which is responsible for the upregulation of cartilage degradation mediated by MMPs.

#### 4.2.3. DHA and MMP-13 Expression

In light of these data and DHA's anti-inflammatory action, Wang et al. conducted a study on the effects of IL-1*β* on human chondrosarcoma SW1353 cells and on a rat model of adjuvant-induced arthritis (AIA), in order to test for DHA's possible protective effects on OA [46]. First, using flow cytometry and an MTT (3-(4,5-dimethylthiazol-2-yl)-2,5-diphenyltetrazolium bromide) assay, DHA concentrations <50 *μ*g/mL were established as being safe for cell treatment. The mRNA and protein expressions were evaluated by RT-qPCR, ELISA, and Western blotting. Studies conducted on SW1353 cells treated with increasing DHA concentrations reported that DHA inhibits both MMP-13 mRNA expression and IL-1*β*-enhanced MMP-13 protein expression in a dose-dependent manner, with a maximal effect at a DHA concentration equal to 50 *μ*g/mL. The mechanism underlying this inhibitory effect was also evaluated, concluding that DHA has no effect on JNK or ERK1/2 activation. On the contrary, DHA inhibits IL-1*β*-induced p38 activation, and particularly, p-38 phosphorylation. *In vivo* studies in an AIA rat model confirmed the results obtained in cells pertaining to MMP-13 expression. In particular, histological and immunohistochemistry analyses have shown that the treatment of AIA rats with DHA increases the thickness of articular cartilage, reducing its destruction by inhibiting MMP-13 expression in the cartilage matrix. Many *in vitro* and *in vivo* studies have demonstrated the positive effects of n-3 PUFAs on cartilage repair, but these need to be confirmed by further clinical trials [47].

#### 4.2.4. DHA, EPA, and Apoptosis

OA is also characterized by joint marginal osteophyte generation accompanied by synovitis. Therefore, OA was induced in the human chondrosarcoma cell line SW1353 by inflammatory factor IL-1*β* stimulation, and the effect of DHA on cell apoptosis was evaluated [48]. By determining B-cell lymphoma-2 (Bcl-2), Bcl-2-associated X protein (Bax), and cleaved caspase-3 expression, in addition to ERK, JNK, and p38 MAPK, in both the presence and absence of DHA, it was established that DHA has an inhibitory effect on IL-1*β* and reduces apoptosis by inhibiting the activation of the MAPK signaling pathway. In addition, EPA, injected intra-articularly into a mouse model of OA showed an antiapoptotic effect on chondrocytes in the presence of oxidative stress [7].

#### 4.2.5. Controlled-Release EPA

A new therapeutic strategy for OA was investigated, in which patients could be treated with controlled-release EPA [49]. The study model was that of male mice exhibiting destabilization of the medial meniscus (DMM), treated with either a single injection of EPA (3 mg/mL) or with gelatin hydrogels containing EPA (3 mg/mL). Histological evaluation was carried out at 1 week and 8 weeks after DMM surgery. Gelatin hydrogels represent a safe drug delivery system through which a physiologically active substance, such as EPA, is gradually released in less than three weeks. Moreover, in this study, immunohistochemical analyses showed that EPA, incorporated into the gelatin hydrogel and released slowly over time, improved MMP-3-, MMP-13-, IL-1*β*-, and p-IKK *α*/*β*-positive cell ratios more effectively than a single injection of EPA, thereby grounding a more effective response to OA progression. This could represent an innovative and more efficient treatment for OA patients.

### 4.3. OA, Obesity, and PUFAs

Although obesity is considered one of the risk factors for knee OA given the increased load on bearing joints, it is also associated with OA in low-load-bearing joints, such as hand joints. Therefore, it has been shown that excess adipose tissue has not only a biomechanical action but also alters cartilage metabolism through the release of cytokines and adipokines. In this process, elevated leptin levels appear to play a particularly important role [50–52]. Furthermore, lipid-lowering drugs may modify the lipid profile and OA progression [50]. It is known, in fact, that in obesity, a diet richer in saturated fatty acids (SFA) and n-6 PUFAs determines an increase in systemic inflammation; while a higher n-3 PUFAs intake reduces joint degeneration with a strong anti-inflammatory and antioxidant effect [51, 53]. In particular, in a study conducted on porcine cartilage explant and human articular chondrocytes (HACs), it was shown that a leptin concentration equal to 10 *μ*g/mL, alone or in combination with IL-1*β*, determines cartilage destruction, with NF-*κ*B, ERK, JNK, and p38 activation and consequent MMP3, MMP13, and ADAMTS4 secretion [52]. Treatment with EPA and DHA had protective effects; it inhibited cartilage damage following reduced ADAMTS4 secretion due to lower NF-*κ*B and JNK activation.

#### 4.3.1. PUFAs and Posttraumatic Osteoarthritis (PTOA)

The fact that n-6 and n-3 PUFAs' contents in the diet influence OA onset in obesity has also been verified in posttraumatic osteoarthritis (PTOA) [54]. This is typically a disease that arises following trauma to the joints, and it presents obesity and metabolic syndrome as risk factors. Using the fat-1 transgenic mouse model, the n-6/n-3 PUFAs ratio was genetically altered through the n-3 fatty-acid desaturase enzyme, encoded by fat-1 gene, the function of which is dehydrogenating n-6 to n-3 PUFAs. In particular, male and female fat-1 and wild-type (WT) mice with PTOA induced by DMM surgery were fed a diet rich in n-6 PUFAs. The results obtained demonstrate that the increased serum content of n-3 PUFAs in obese fat-1 mice reduced OA and synovitis, despite their having a similar body weight to that of WT mice. Furthermore, this reduction is sex- and diet-dependent. In fat-1 mice, a reduction in systemic inflammation was also recorded; in fact, the greater presence of n-3 PUFAs in serum is related to a decrease in proinflammatory cytokines, such as interferon-*γ* (IFN-*γ*), TNF-*α*, and monocyte chemoattractant protein-1(MCP-1), and an increase in the anti-inflammatory response. Therefore, OA severity under obese conditions is more related to serum fatty acid levels and composition than to body weight. This study demonstrates that joint damage can be reduced in obese subjects given the greater presence of circulating n-3 PUFAs compared to n-6.

#### 4.3.2. Lipid Profile and OA

A different balance between the FAs taken with the diet can alter the phospholipid layer that covers the cartilage surface [53]. This layer offers protection during joint loading, and therefore, protection against OA onset and severity. In order to evaluate the serum and synovial fluid lipid composition under OA conditions, a study was conducted on male mice fed for 24 weeks on a low-fat control diet and a high-fat diet (HFD) rich in SFA, n-3 PUFAs, or n-6 PUFAs. OA was induced following surgery for DMM. The results obtained show that mice fed a diet rich in n-3 PUFAs have a more stable lipid composition in the synovial fluid than mice fed a diet rich in SFAs and n-6 PUFAs. Furthermore, in serum, n-3 PUFAs negatively correlate with OA severity and positively correlate with adiponectin, which triggers an anti-inflammatory response. A contrary condition is associated with n-6 PUFAs, which also have a positive correlation with inflammatory adipokines. In addition, in serum, a high n-3/n-6 PUFA ratio corresponds to a less severe OA condition, with a lower presence of inflammatory adipokines. In contrast, the lipid profile in the synovial fluid of the joints was evaluated in a study conducted on patients with end-stage knee OA and nonsymptomatic controls [50]. Mass spectrometry was used to measure SFAs, monounsaturated fatty acids (MUFAs), and n-6 and n-3 PUFA concentrations; the n-6/n-3 ratio was also defined. In the synovial fluid of subjects with OA, the SFA concentrations, in particular tetracosadienoic acid, and the MUFA concentrations, in particular nervonic acid, were higher than in nonsymptomatic subjects. In contrast, the n-6 concentration, especially arachidonic acid, was lower in OA patient fluid than in the control, while the n-3 concentration was comparable. Therefore, the PUFA profiles in the two groups show a lower n-6/n-3 ratio in the OA group compared to the control. The results obtained in this study appear to contrast with other research, in which OA patients showed a higher content of n-6 PUFAs and a consequently higher n-6/n-3 ratio, explained by the proinflammatory properties of n-6 PUFAs and their derivatives. The contrast between the results of this study and those of previous ones can be attributed to the different OA phenotypes considered in this study, and the lower number of patients (only 29), whose lifestyle and pharmacological history were not known. Additionally, in this study, patients had end-stage knee OA with total knee replacement. In this condition, synovitis and inflammation may be replaced by a more profibrotic phase, in accordance with a lower content of n-6 PUFAs, the concentration of which may be a little higher in the initial stages of OA. A 48-month study was conducted on 2092 subjects with knee OA [55]. The intake effects of total fat, SFAs, MUFAs, and PUFAs on knee OA progression were assessed annually. In particular, OA progression was monitored by evaluating over time the reduction in quantitative joint space width (JSW) between the medial femur and tibia of the knee, depending on the intake of the various types of fat. This study depends on the knowledge that a diet rich in fat, and the consequent overweight condition, determines variations in joint loading and changes in cartilage, highlighting a strong correlation between diet and OA pathogenesis. Therefore, it was established that total fat and SFAs are associated with a JSW loss, and thus, with greater knee OA structural progression. In contrast, subjects on a diet rich in MUFAs and PUFAs display lower JSW loss with slower knee OA progression. All this demonstrates that dietary fat is closely associated with knee OA structural progression.

#### 4.3.3. n-3 PUFAs and GPR 120

It is known that OA onset and progression in obesity are more related to lipid metabolism homeostasis and circulating adipokines than to adiposity and body weight. In obesity, oxidative stress determines adipocyte lipolysis, with the consequent release into circulation of free fatty acids, which can generate pro- or anti-inflammatory molecules [56]. For example, binding between n-3 PUFAs and G-protein coupled receptor 120 (GPR 120) leads to the synthesis of protectins and resolvins, thereby mediating anti-inflammatory effects in different cell types [5, 56]. In this regard, a study was conducted to define GPR120 involvement in OA [56]. GPR120 and its agonist DHA are already known to confer protection in obesity-associated type 2 diabetes. GPR120's involvement in cartilage degeneration during OA progression was confirmed by studies conducted in GPR120 knockout mice with OA surgically induced by ACLT. In the same study, the anti-inflammatory effect of GPR120 activation with DHA was reported *in vitro* in human chondrocytes. Furthermore, *in vivo* studies on OA have shown that n-3-GPR120 signaling inhibition leads to alterations in bone remodeling and osteophyte formation of the subchondral bone.

### 4.4. Pain in OA and PUFAs

Furthermore, n-3 PUFA supplementation in humans also causes a reduction in pain and structural damage associated with increased function [57]. Some clinical studies have been performed on patients with active OA whose daily diet was supplemented with fish oil [38]. The trials ranged in duration from 12 to 104 weeks, and the number of patients enrolled ranged from 81 to 202. In these trials, WOMAC (Western Ontario and McMaster Universities Arthritis Index) scores were found to be lower, accompanied by a reduction in pain and a lower use of NSAID/analgesic. Importantly, none of these studies had any adverse effects related to the use of fish oil.

#### 4.4.1. n-6:n-3 PUFA Ratio, Pain, and Psychosocial Distress

Knee OA is known to be characterized not only by joint-specific inflammation but also by systemic inflammation [58]. Additionally, people with OA present chronic pain, functional limitations, and psychosocial suffering. Therefore, a study was conducted on 167 patients with knee OA in order to verify the possible effects of PUFAs on various OA aspects. In particular, a pilot study was conducted on the n-6:n-3 PUFA ratio in OA, taking into account the known anti-inflammatory, antinociceptive, and psychosocial distress ameliorative effects of n-3 PUFAs, compared to the opposite effects of n-6 PUFAs. To do this, the participants' plasma was collected, and the n-6:n-3 PUFAs ratio was measured. The data obtained were also evaluated on the basis of the clinical and functional importance of the n-6:n-3 PUFA ratio, the recommended value of which varies from 2 : 1 to 5 : 1. The group with a low n-6:n-3 ratio suffered less knee pain and had better physical functioning and less psychosocial distress.

#### 4.4.2. EPA, L-Serine, and Pain

Additionally, a randomized, double-blind, placebo-controlled, parallel group study was recently conducted to evaluate the effect of EPA and L-Serine on lower-back and knee pain [59]. The study was conducted on 120 adult participants with lower-back and knee pain for at least three months, divided into a placebo group and a group given 594 mg of L-Serine and 149 mg of EPA daily for eight weeks. In the following four weeks of observation, an improvement in lumbar and knee pain was recorded in subjects treated with the combination of L-Serine, which acts on nerve function, and EPA, which exerts an anti-inflammatory effect. The results obtained here will be further verified in future clinical studies.

### 4.5. Maresins and Resolvins in OA Treatment

#### 4.5.1. Anti-Inflammatory Effect of Maresin-1

In order to verify the possible anti-inflammatory therapeutic effect of maresin-1, a metabolite of DHA, a study was conducted in rats with OA, whereby endogenous maresin-1 was measured in the intra-articular lavage fluid (IALF) of rats in single-session treadmill experiments [60]. In another group of rats, OA was induced by monosodium iodoacetate (MIA) in the presence of exogenous maresin-1. Finally, the possible action mechanisms of maresin-1 were examined in IL-1*β*-induced rat FLSs. ELISA showed that 4 hours after exercise, the maresin-1 levels in IALF were higher, while in the MIA model of OA, there was an increase in COL2A1 in the cartilage and a reduction in MMP-13 in the synovium. *In vitro* experiments also recorded MMP-13 reductions, indicating PI3k/Akt (phosphatidylinositol 3-kinase/protein kinase B) signaling pathway activation and NF-*κ*B p65 signaling pathway inhibition as action mechanism of maresin-1.

#### 4.5.2. Anti-Inflammatory, Antiapoptotic, and Antioxidant Role of RvD1

D resolvins (RvD) are other DHA derivatives [61]. In particular, RvD1's anti-inflammatory, anticatabolic, and antiapoptotic properties were tested in human OA chondrocytes treated with IL-1*β* or HNE (4-hydroxynonenal). It is known from previous dog OA experiments that the level of RvD1 increases in the synovial fluids of dogs to resolve inflammatory conditions, but it has also been shown that RvD1 can regulate signaling pathways involved in oxidative stress and cell death. Research on OA chondrocytes has shown that RvD1 inhibits both mRNA transcription and COX-2, iNOS, and MMP-13 protein expression. It has been demonstrated that RvD1 deactivates the p38/MAPK, JNK 1/2, and NF-*κ*B p65 signaling pathways, reducing IL-1*β*-induced inflammation in OA. Furthermore, the synthesis of PGE2 and NO is reduced, the accumulation of which is involved in OA progression and pain. In this experimental model, the involvement of apoptosis and oxidative stress in OA and RvD1's possible protective role was also investigated. Thus, apoptosis was induced by HNE treatment in OA chondrocytes pretreated with RvD1. The reported results confirm RvD1's antiapoptotic function, which would prevent damage to cartilage. In fact, RvD1 inhibited the HNE-induced activation of caspase-3 and -9, as well as LDH (lactate dehydrogenase) release, but it also increased antiapoptotic protein Bcl2 expression and reactivated Akt. The antioxidative role of RvD1 was demonstrated as the GSH (glutathione) pool was increased in OA chondrocytes. Therefore, supplementation with RvD1 can improve lesions and cartilage degradation, not only reducing the inflammatory state in OA but also preventing oxidative stress-induced apoptosis in OA chondrocytes, as RvD1 is able to restore redox status.

#### 4.5.3. Association between Macrophages and RvD1

Additionally, in studying the relationship between OA, metabolic syndrome, and obesity, the roles of macrophage infiltration in the joint synovium and of RvD1 were investigated [62, 63]. The association between macrophages and RvD1 derives from the fact that, in inflamed tissues, resolvins mediate the transition of macrophages from a proinflammatory (M1) to an anti-inflammatory (M2) state; it also derives from the role that macrophage polarization plays in obesity-induced OA [62]. RvD1 could reduce this pathology by changing the macrophage proinflammatory condition. In order to verify this, C57Bl/6 mice were fed an HFD, and PTOA was induced by surgical destabilization of the meniscus. Under these conditions, it has been established that an HFD diet alone does not worsen cartilage degradation, whereas posttraumatic OA does. However, it has been found that an HFD worsens OA synovitis, resulting in greater macrophage infiltration, even in the absence of a posttraumatic event. Inflammation induced by obesity under OA conditions is determined by both the migration and the local proliferation of synovial macrophages. Under these conditions, the role of macrophages has been confirmed by the intra-articular injection of clodronate liposomes, whose function is to analyze the effects of these cells. The resulting macrophage depletion improves synovitis and cartilage destruction in the obese mice model with PTOA. Once the role of macrophages in obesity-induced OA progression was verified, the effect of treatment with RvD1 was evaluated. RvD1's presence improves synovium thickening, as it changes the macrophages' gene expression from a proinflammatory condition to an anti-inflammatory state. Furthermore, under obese conditions, intra-articular RvD1 administration limits cartilage degradation by polarizing M2 macrophages. Moreover, randomized clinical trials have demonstrated a reduction in musculoskeletal pain in the presence of n-3 PUFAs, both in subjects with knee OA and in subjects with exercise-induced pain [64]. The effects of n-3 PUFAs on pain are attributable to cartilage degradation reduction and the increase in resolvins in synovial fluids.

## 5. Discussion and Conclusions

**Figure 1:**
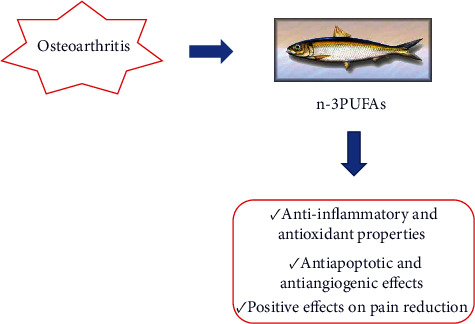
Main properties of n-3 PUFAs in OA treatment.

OA, considered by the Osteoarthritis Research Society International (OARSI) a disease characterized by molecular, anatomical, and physiological alterations, needs effective treatment with fewer side effects than current therapies [65]. Therefore, it has been shown that dietary interventions can yield positive results in preventing and slowing the disease. In this context, for example, polyphenols, thanks to their antioxidant and anti-inflammatory properties, are effective. In particular, Valsamidou et al. reported preclinical and clinical studies attesting the positive role of combined polyphenols in the OA treatment [65]. In this review, however, we wanted to focus on the latest research related to the treatment of OA with other components of the diet, PUFAs. *In vitro* and *in vivo* studies, as well as studies on patients, have shown that nutraceuticals such as n-3 PUFAs can prevent and counteract joint degeneration and cartilage loss in OA. Their efficacy is demonstrated at various molecular levels, but their anti-inflammatory and antioxidant properties seem to have a greater chondroprotective role. In addition, clinical trials have also reported their positive effect on the reduction of pain associated with OA, which is very important as it could improve patients' quality of life (Table 1) (Figure 1).

## Figures and Tables

**Table 1:** Effects of PUFAs and derivatives in OA.

First author, year	References	Treatment	Properties	*In vitro*/*in vivo* models	Clinical trials
Xie et al., 2019	[1]	DHA	↓ bone mass loss ↓ osteoclast differentiation ↓expression of TRAP and CTSK ↓ osteoclasts differentiation and bone remodeling ↓ RANKL ↓ levels of NFATc1 and MITF ↓ angiogenesis ↓ VEGF–VEGFR2 signaling pathway	ACLT rat OA model; RAW264.7 cells; HUVECs cells	

Yu et al., 2015	[45]	LA/ALA	↓ MMP-13 and IL-1 ↓ paw swelling rate and cartilage damage	Inflammatory human chondrocytes; Sprague-Dawley rats with arthritis induced by Freund's complete adjuvant	

Wang et al., 2016	[46]	DHA	↓ MMP-13 ↓ IL-1*β*-induced p38 activation ↑ thickness of articular cartilage	Human chondrosarcoma SW1353 cells; rat model of AIA	

Xu et al., 2019	[48]	DHA	↓ apoptosis ↓ activation of the MAPK signaling pathway	Human chondrosarcoma SW1353 cells	

D'Adamo et al., 2020	[7]	EPA	↓ apoptosis in the presence of oxidative stress	Mouse model of OA	

Phitak et al., 2018	[52]	EPA and DHA	↓ cartilage damage ↓ ADAMTS4 secretion ↓ NF-*κ*B and JNK activation	Porcine cartilage explant and HACs	

Kimmerling et al., 2020	[54]	n-6 PUFAs	↑ serum content of n-3 PUFAs ↓ OA and synovitis ↑ body weight ↓ IFN-*γ*, TNF-*α*, and MCP-1 ↑ anti-inflammatory response	Fat-1 transgenic mouse model with PTOA induced by DMM surgery	

Wu et al., 2017	[53]	HFD rich in SFA, n-3 PUFAs, or n-6 PUFAs.	↑ n-3 PUFAs, ↓ OA severity, ↑ anti-inflammatory response	Mouse model of OA induced by DMM surgery	

Lu et al., 2017	[55]	Diet rich in total fat, SFAs, MUFAs, and PUFAs	↑ total fat and SFAs ↓ JSW ↑ knee OA progression ↑ MUFAs and PUFAs ↑ JSW ↓ knee OA progression		Subjects with knee OA

Chen et al., 2018	[56]	DHA	↑ GPR120 activation ↑ anti-inflammatory effects	GPR120 knockout mice with OA induced by ACLT; human chondrocytes	OA patients

Akbar et al., 2017	[38]	Diet supplemented with fish oil	↓ WOMAC scores ↓ pain ↓ use of NSAIDs/analgesic		OA patients

Sasahara et al., 2020	[59]	L-serine and EPA	↓ lumbar and knee pain		Patients with lower-back and knee pain

Lu et al., 2020	[60]	Maresin-1	↑ COL2A1 ↓ MMP-13 ↑ PI3k/Akt signaling pathway activation ↓ NF-*κ*B p65 signaling pathway	Rat model of OA induced by MIA; IL-1*β* -induced rat FLSs	

Benabdoune et al., 2016	[61]	RvD1	↓ COX-2, iNOS, MMP-13 ↓ p38/MAPK, JNK 1/2, and NF-*κ*B p65 signaling pathways ↓ PGE2 and NO ↓ pain ↓ activation of caspase-3 and -9 ↓ LDH release ↑ Bcl2 and AKT ↑ GSH pool ↓ oxidative stress-induced apoptosis	Human OA chondrocytes treated with IL-1*β* or HNE	

Sun et al., 2019	[62]	RvD1	↑ macrophages' gene expression from a proinflammatory condition to an anti-inflammatory state ↓ cartilage degradation	Obese mice model with PTOA induced by DMM surgery	

↑ increase, ↓ decrease. DHA: docosahexaenoic acid; ACLT: anterior cruciate ligament transection; OA: osteoarthritis; RAW264.7: mouse mononuclear macrophage leukemia cells; HUVECs: human umbilical vein endothelial cells; TRAP: tartrate-resistant acid phosphatase; CTSK: cathepsin K; RANKL: receptor activator of nuclear factor kappa-*Β* ligand; NFATc1: nuclear factor of activated T-cells, cytoplasmic 1; MITF: microphthalmia-associated transcription factor; VEGF: vascular endothelial growth factor; VEGFR2: VEGF receptor 2; LA: linoleic acid; ALA: *α*-linolenic acid; MMP-13: matrix metalloproteinase-13; IL-1*β*: interleukin-1*β*; AIA: adjuvant-induced arthritis; MAPK: mitogen activated protein kinase; EPA: eicosapentaenoic acid; ADAMTS4: disintegrin and metalloproteinase with thrombospondin motifs-4; NF-*κ*B: nuclear factor-*κ*B; JNK: c-Jun N-terminal kinases; HACs: human articular chondrocytes; n-6,-3 PUFAs: n-6,-3 polyunsaturated fatty acids; IFN-*γ*: interferon-*γ*; TNF-*α*: tumor necrosis factor-*α*; MCP-1: monocyte chemoattractant protein-1; PTOA: posttraumatic osteoarthritis; DMM: destabilization of the medial meniscus; HFD: high-fat diet; SFA: saturated fatty acids; MUFAs: monounsaturated fatty acids; JSW: joint space width; GPR120: G-protein coupled receptor 120; WOMAC: western Ontario and McMaster universities arthritis index; NSAIDs: nonsteroidal anti-inflammatory drugs; COL2A1: type II collagen; PI3K/Akt: phosphatidylinositol 3-kinase/protein kinase B; MIA: monosodium iodoacetate; FLSs: fibroblast-like synoviocytes; RvD1: resolvin D1; COX-2: cyclooxygenase-2; iNOS: inducible nitric oxide synthase; p38/MAPK: p38/mitogen activated protein kinase; NF-*κ*B p65: nuclear factor-*κ*B p65 subunit; PGE2: prostaglandin E2; NO: nitric oxide; HNE: 4-hydroxynonenal; LDH: lactate dehydrogenase; Bcl-2: B-cell lymphoma-2; GSH: glutathione.
